# Performance of Dry Powder Inhalers with Single Dosed Capsules in Preschool Children and Adults Using Improved Upper Airway Models

**DOI:** 10.3390/pharmaceutics6010036

**Published:** 2014-02-06

**Authors:** Sandra Lindert, Antje Below, Joerg Breitkreutz

**Affiliations:** 1 Institute of Pharmaceutics and Biopharmaceutics, Heinrich-Heine University, Duesseldorf 40225, Germany; E-Mail: Sandra.Lindert@hhu.de; 2 Rottendorf Pharma GmbH, Ennigerloh 59320, Germany; E-Mail: Antje.Below@rottendorf.com

**Keywords:** dry powder inhaler, single dosed capsules, upper airway model, preschool children, Cyclohaler, Handihaler, Spinhaler, *in vitro* testing

## Abstract

The pulmonary administration of pharmaceutical aerosols to patients is affected by age-dependent variations in the anatomy of the upper airways and the inhalation pattern. Considering this aspect, different upper airway models, representing the geometries of adults and preschool children, and a conventional induction port according to the European Pharmacopeia were used for *in vitro* testing of dry powder inhalers with single dosed capsules (Cyclohaler^®^, Handihaler^®^ and Spinhaler^®^). Deposition measurements were performed using steady flow rates of 30 and 60 L/min for the Handihaler^®^/Spinhaler^®^ and 30, 60 and 75 L/min for the Cyclohaler^®^. The inhalation volume was set at 1 L. For the Cyclohaler^®^, the *in vitro* testing was supplemented by a pediatric inhalation profile. Slight differences of pulmonary deposition between the idealized adult (11%–15%) and pediatric (9%–11%) upper airway model were observed for the Cyclohaler^®^. The applied pediatric inhalation profile resulted in a reduction of pulmonary deposition by 5% compared to steady conditions and indicated the influence of the inhalation pattern on the amount of pulmonary deposited particles. The comparison of two pediatric upper airway models showed no differences. The performance of the Handihaler^®^ was similar to the Cyclohaler^®^. The Spinhaler^®^ showed an insufficient performance and limited reproducibility in our investigations.

## 1. Introduction

Diseases of the respiratory tract are very common, especially in industrialised countries. Bronchial asthma, manifested in typical symptoms such as coughing and wheezing caused by airway obstruction, is one of the most frequent diseases occurring in the first years of life. However, the prevalence of asthma in children is much higher compared to adults [1,2]. In the first instance, the therapy demands a pulmonary administration to avoid systematic side effects of the active pharmaceutical ingredients (API). In general, two main routes of pulmonary administration can be distinguished: nasal and oral inhalation. Regarding age, physiological features and mental capacity, the nasal route is the option of choice in the treatment of newborns, infants and older children up to the age of 4, by using facemasks and nebulisers as delivery devices [3]. Recently published data described the use of high-flow nasal cannula for the aerosol delivery to children [4]. The oral administration of aerosols is principally limited to preschool children (mainly by using valved holding chambers), older children and adults, while dry powder inhalers (DPI) or metered dose inhalers (MDI) are usually applied as drug delivery systems in these age groups [3]. The preschool pediatric population that this study focused on is defined by a range of age between 2 and 5 years. The delivery of pharmaceutical aerosols via DPIs is the preferred method in pulmonary therapy for this age group [5]. During oral administration, the inhalable API has to overcome a number of anatomical barriers to reach the lung as target organ. In the oral cavity, the inhaled drug particles reach the pharynx, where they are subject to a strong change of direction caused by the throat geometry. Subsequently, the API particles are transported via the larynx and the trachea to the target site of the peripheral airways. In the case of bronchial asthma, the most prescribed medications are inhalable β_2_-sympathomimetics, such as salbutamol sulfate, which has been used in this study. This API acts as agonist at β_2_-receptors of smooth muscles located in the whole lung and especially in the peripheral airways, the bronchioles [6]. Thus, the structure and physiology of the upper and peripheral airways are essential aspects in the field of respiratory diseases. In this context, the main factors influencing the delivery of pharmaceutical aerosols to patients of all ages need to be taken into consideration. These factors can be generally divided into three factors: the patient, the drug delivery device and the administered formulation [7]. The patient’s inter- and intra-individual variability of the upper airway geometry is related to age and can be described as the most crucial factor in pulmonary drug delivery [8]. 

In childhood, the lung is not yet fully developed and several differences between children and adults can be observed [9]. The pharynx, larynx and trachea are smaller in children than in adults, while other parts, like the tongue related to the entire oral cavity, are even more expanded compared to adult dimensions [10]. In addition to the anatomical differences, the peak inspiratory flow rate (PIF) and the inhalation volume of children are lower than in adults, but the inhalation time is shorter compared to an adult inhalation process [11,12]. The capability of preschool children to reach sufficient flow rates increases with age [13]. However, the inhalation pattern differs in children with acute asthma [14] and plays an important role to ensure an adequate treatment of respiratory diseases. In case of DPIs, the patient has to provide sufficient flow rates to induce the deaggregation of the powder mixture (separation of micronized API particles from carrier particles). The required energy for this process has to be supplied by the inhalation force (high PIF and inhalation volume) of the patient [15] and depends on the respective DPI, which is another important factor. 

The maximum flow rate generated by the patient is affected by the resistance of the device, which is closely linked to the structure and mechanism of deaggregation. The higher the resistance of the DPI, the higher is the force needed to ensure a sufficient pressure drop within the device and the lower the maximum flow rate [16]. 

Considering that DPIs are described as appropriate delivery devices for children of preschool age and older [3], there is a need for systematical investigations of the influencing parameters given above. On the one hand, costly *in vivo* studies including scintigraphic images using radioactive labeled material or pharmacokinetic studies of absorption and elimination can be carried out [17]. On the other hand, the performance of *in vitro* studies is desirable to assess the aerodynamic particle size distribution of aerosols and to get information about the pulmonary deposition of the API. Standard methods to determine the deposition pattern and the inhaler performance described in the European Pharmacopeia (induction port, preseparator, 4 kPa pressure drop and 4 L volume) poorly reflect *in vivo* conditions and serve solely as quality control tool. Preliminary *in vitro* investigations showed a discrepancy of the deposition data in adults and children [18]. In the particular case of children, there is a lack of sufficient *in vivo* data to verify results produced by *in vitro* studies. Regarding an enhanced *in vitro* and *in vivo* correlation, physical upper airway models representing the anatomy of different age groups in more detail can be used to determine the particle deposition. 

**Figure 1:**
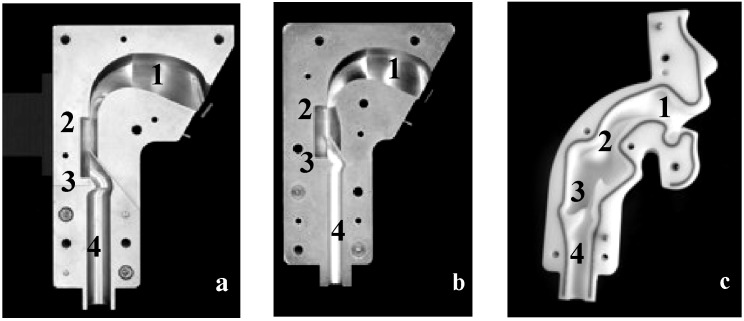
Physical upper airway models including the different regions 1, oral cavity; 2, pharynx; 3, larynx; and 4, trachea. (**a**) idealized adult upper airway model; (**b**) idealized pediatric upper airway model representing 4–5 years old children; and (**c**) realistic pediatric upper airway model representing 4–5 years old children.

A number of upper airway models utilized for several age groups are described in the literature [19,20]. For instance, the idealized adult upper airway model (“Alberta throat”, Figure 1a) [21] is widespread in the field of inhaler performance testing and showed good agreement with *in vivo* data. For pediatric use, the majority of the described upper airway models represent premature infants [22] and newborns [23]. Physical upper airway models applicable for older children are comparatively rarely described [10,24,25,26]. Golshahi *et al.* [24] recently introduced a child throat imitating the geometry of children at the age of 6–14 years. This is the main difference between the idealized pediatric upper airway model [10] (Figure 1b) and the pediatric realistic upper airway model [25] (Figure 1c) representing the age group of 4–5 years old children, used in this study. Both investigated upper airway models were developed based on MRI data and represent children from 4–5 years of age. Preliminary experiments using passive multidose DPIs (Easyhaler^®^ and Novolizer^®^) exhibited, that the idealized pediatric upper airway model leads to reproducible results of inhaler performance and it is recommended as a feasible implement for DPI testing [27]. 

This study focuses on *in vitro* performance testing of reusable DPIs with disposable single dosed capsules (Cyclohaler^®^, Handihaler^®^ and Spinhaler^®^) using improved upper airway models representing adults and preschool children compared to a conventional impactor inlet. The inhalation parameters flow rate and volume were varied (steady flow rates) and, in the case of the Cyclohaler^®^, a time-varying inhalation profile was used to determine the particle deposition in the different oropharyngeal and pulmonary compartments. 

## 2. Experimental Section

### 2.1. Materials

#### 2.1.1. Dry Powder Inhalers

Three dry powder inhalers were investigated: Cyclohaler^®^ (Cyclocaps Salbutamol^®^, PB Pharma, Meerbusch, Germany), Handihaler^®^ (Spiriva Handihaler^®^, Boehringer Ingelheim, Ingelheim, Germany) and Spinhaler^®^ (Intal^®^, Fisons Arzneimittel, Cologne, Germany). The devices differ in their mechanism of powder deaggregation caused by the construction of the inhaler and the airflow resistance. Whereas Cyclohaler^®^ and Handihaler^®^ contain a fine grid to disperse the powder particles; the Spinhaler^®^ is equipped with a propeller, which is operated by the air stream of the patient. With an airflow resistance of 0.055 (cm H_2_O)^0.5^ l min^−1^ for the Cyclohaler^®^ [28] and 0.051 (cm H_2_O)^0.5^ l min^−1^ for the Spinhaler^®^ [16], they belong to the low resistant dry powder inhaler. In contrast, the Handihaler^®^ (0.158 (cm H_2_O)^0.5^ l min^−1^) is counted among the group of high resistant DPIs [29]. The present studies were performed using capsules from the commercially available product Cyclocaps Salbutamol^®^ to eliminate the influence of the powder formulation on the deposition pattern. Finally, the native capsule piercing mechanism of the Cyclohaler^®^ could not be ensured for the Handihaler^®^ and the Spinhaler^®^, which was a limitation of our study. The capsules contain salbutamol sulfate as API and α-lactose monohydrate as drug carrier in an interactive mixture. 

#### 2.1.2. Upper Airway Models

In addition to the conventional setup according to European Pharmacopeia consisting of a 90° induction port and a preseparator [30], three physical upper airway models were tested. An idealized adult upper airway model [21], an idealized pediatric upper airway model [10] and a realistic pediatric upper airway model [25] were compared (Figure 1). These physical upper airway models consider the anatomical dimensions of the oral cavity, pharynx, larynx and trachea, which were determined on the basis of NMR (Magnetic Resonance Spectroscopy) and CT (Computed Tomography) data of adults and children of the respective age group. For the realistic pediatric model, data of 11 children at the age of 4–5 years and for the idealized pediatric model MRT scans of 5 years old children were used to calculate the dimensions of the different regions located in the upper airways.

### 2.2. Methods

#### 2.2.1. Time-Varying Inhalation Profile

**Figure 2:**
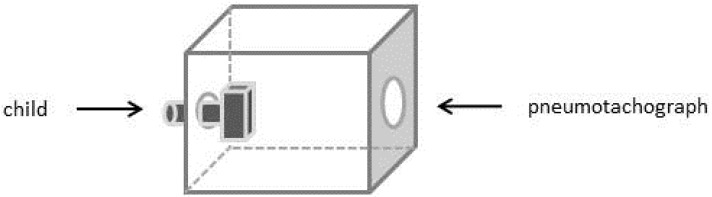
Schematic presentation of the experimental setup for the generated time-varying inhalation profile; on the **left** side the pediatric patient; in the **middle** the Cyclohaler^®^ incorporated in an air tight plastic box; on the **right** side the pneumotachograph.

In order to get preliminary information about inhalation parameters of the patient using the Cyclohaler^®^, a time-varying inhalation profile was recorded during the inhalation through an empty device by a 4 years old male child (height of 110 cm and body weight of 20 kg). The boy was guided to inhale in an upright position in accordance to handling instructions of the marketed product. For this experiment, the inhaler was placed in an air tight plastic box, which had an associated pneumotachograph (Masterscope Viasys/Jaeger, Germany) on one side. On the opposite side, the inhaler mouth piece was accessible for the child (Figure 2). The peak inspiratory flow rate (PIF) and the inhalation volume over the time were recorded.

#### 2.2.2. Deposition Measurements—Steady Flow Rates

According to the standard method of the European Pharmacopeia (Ph. Eur. 2.9.18) for the aerodynamic assessment of fine particles, the experimental setup consisted of a next generation impactor (NGI, Copley Scientific Limited, Nottingham, UK) including a conventional inlet and preseparator. A flow meter (TSI 4000, TSI, Aachen, Germany) and a vacuum pump were used to provide a steady flow rate. The inhalation volume was adjusted by the process time to each flow rate. 

**Figure 3:**
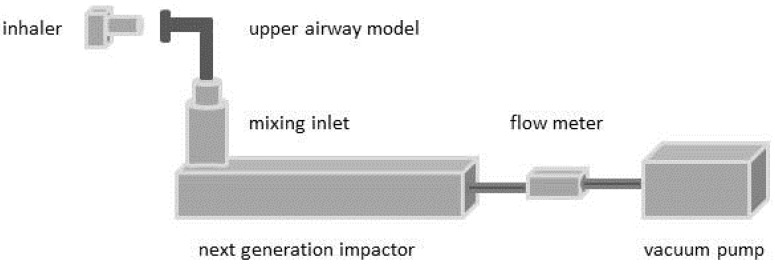
Schematic presentation of the experimental setup used for deposition measurements.

For further experiments the inlet and preseparator described in Ph. Eur. were replaced by the physical upper airway models, which were connected to the NGI by a mixing inlet (Figure 3). The inner surfaces of the upper airway models and the cups of the NGI were coated by a 3% (*w*/*w*) solution of Brij^®^ (Serva Eletrophoresis, Heidelberg, Germany) and glycerol to avoid a possible resumption of deposited particles in the airstream and to consider the wet conditions in the mucosal environment [27]. Ten capsules were used for each experiment. The deposited salbutamol sulfate was collected and quantified by high performance liquid chromatography (HPLC). The results were assessed based on the oropharyngeal deposition as amount of API collected in the upper airway model (including the inlet and preseparator according to Ph. Eur.), the pulmonary deposition as particles smaller than 5 μm and the emitted dose. The pulmonary deposition was calculated using the cut off diameters of the respective flow rate [30].

The performance of the Cyclohaler^®^ was investigated using three different steady flow rates (30, 60 and 75 L/min) and all described upper airway models (except the realistic pediatric model). The inhalation volume was adjusted at 1 L. The Handihaler^®^ and the Spinhaler^®^ were investigated at two steady flow rates (30 and 60 L/min) using the idealized adult and pediatric model. Table 1 shows the investigated flow rates and the achieved pressure drops within the different devices. 

**Table 1:** Pressure drops of the inhalers at different flow rates using a water column and dosing tube connected to the inhaler; mean ± SD; *n* = 3.

Flow rate [L/min]	Pressure drop [kPa] ± s Cyclohaler^®^	Pressure drop [kPa] ± s Handihaler^®^	Pressure drop [kPa] ± s Spinhaler^®^
30	0.22 ± 0.01	1.59 ± 0.05	0.14 ± 0.01
60	0.94 ± 0.03	6.14 ± 0.02	1.04 ± 0.01
75	1.17 ± 0.02	-	-

#### 2.2.3. Deposition Measurements—Time-Varying Inhalation Profile

The pediatric time-varying inhalation profile was used for *in vitro* deposition studies. The experimental setup (Figure 3) was extended by an electronic lung device (ASL 5000, Ingmar Medical, Pittsburgh, PA, USA), which was connected via a mixing inlet to the NGI. The electronic lung simulated the inhalation profile only inside the DPI and the upper airway model. Within the NGI a constant flow rate of 60 L/min was assured. These deposition measurements were performed using the idealized and realistic pediatric upper airway model and the Cyclohaler^®^ as drug delivery device.

#### 2.2.4. High Performance Liquid Chromatography

The deposition of salbutamol sulfate was evaluated by HPLC (LaChrom Elite, VWR International, Darmstadt, Germany) using a Nucleodur C18 gravity 250/4 (Macherey & Nagel, Dueren, Germany) column [31]. Acetonitrile/acetic acid pH 3 (52/48, *v*/*v*) was chosen as isocratic eluent with a flow rate of 0.45 mL/min. The detection wavelength was set at 276 nm (UV/Vis-detector) and the oven temperature was adjusted at 35 °C.

## 3. Results and Discussion

### 3.1. Time-Varying Inhalation Profile—Experimental Plan

Only limited data regarding *in vivo* inhalation parameters (PIF and inhalation volume) of preschool children are available for the particular inhaler. Bronsky *et al.* [28] described that 91% of 32 children at the mean age of 9.1 years were able to generate flow rates of at least 60 L/min through the Aerolizer^®^, which is quite similar to the Cyclohaler^®^ used in this study. Tiddens *et al.* [32] investigated the inhalation pattern of 96 humans at the age of 6 to 54 years using different types of inhalers (low, medium and high resistant inhalation devices). In the case of low resistant devices, children inhaled a mean volume of only 1.2 L. Both, the described flow rate as well as the inhalation volume do not match the requirements of the Ph. Eur. (2.9.18). 

**Figure 4:**
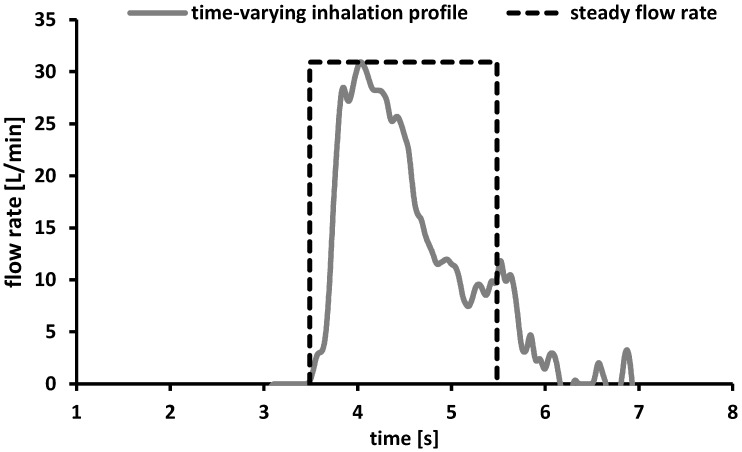
Time-varying inhalation profile of a 4 years old child (grey line) and schematic presentation of a steady flow rate of 30 L/min and an inhalation volume of 1 L expressed as function of the inhalation time (dashed black line).

Based on this lack of information a time-varying inhalation profile of a 4 years old male child was recorded during inhalation through the Cyclohaler^®^ (Figure 4, grey line). The additional information obtained by recording the inhalation profile formed the basis for the experimental plan and the chosen process parameters. 

The child achieved the maximum PIF of 30 L/min within the first 0.46 s while the complete inhalation period took 3.46 s. The inhalation volume calculated as a function of the area under the curve was about 1 L and is comparable to the data described in literature [32]. The maximum PIF of only 30 L/min was lower than the flow rates reported in literature [28] and would result in an insufficient therapy using the Cyclohaler^®^ as drug delivery device [33]. To cover a broad range of possible flow rates, the process parameters for further deposition measurements under steady conditions were chosen as follows. The minimum flow rate was set to 30 L/min to consider the PIF resulted from the time-varying inhalation profile. As maximum flow rate 75 L/min was selected as approach to the required conditions according to the Ph. Eur. and a flow rate of 60 L/min should consider the data provided by literature [28]. On the basis of the results discussed above, the inhalation volume was adjusted at 1 L. 

To get an indication of the differences between steady flow rates and time-varying inhalation profiles, a systematical presentation of a steady flow rate proceed (30 L/min, volume 1 L) is given above (Figure 4). The maximum flow rate was achieved directly after starting the process and formed a plateau until the end of the process (regulated by the adjusted inhalation volume) and subsequently dropped down rapidly.

### 3.2. Deposition Measurements—Steady Flow Rates

In the treatment of respiratory diseases some key parameters have to be evaluated to ensure a sufficient therapy: the emitted dose as amount of API particles leaving the mouth piece, the oropharyngeal deposition and the pulmonary deposition, representing the amount of API, which can theoretically reach the lung and causes the therapeutic effect. The pulmonary deposition is affected by a multitude of factors, like the age-specific anatomy or the flow rate and requires a systematical investigation considered in this study. 

**Figure 5:**
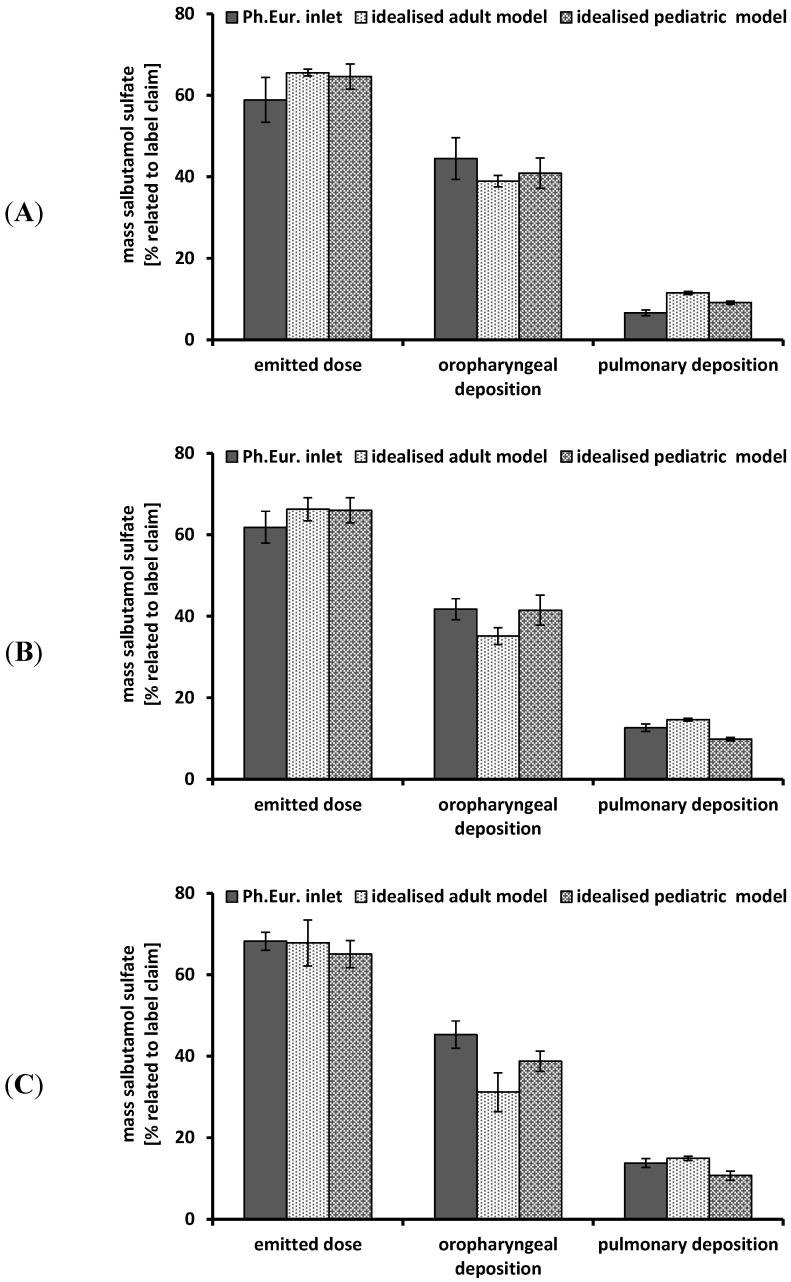
Performance of the Cyclohaler^®^ at different flow rates using different impactor inlets; Ph. Eur. inlet consisting of induction port and preseparator; idealized adult and pediatric upper airway model; realistic pediatric upper airway model. (**A**) flow rate 30 L/min; (**B**) flow rate 60 L/min; (**C**) flow rate 75 L/min; emitted dose, oropharyngeal deposition and pulmonary deposition were related to label claim; mean ± SD, *n* = 3.

Figure 5 includes the results of the *in vitro* deposition testing of the Cyclohaler^®^ using steady flow rates and different impactor inlets. The emitted dose ranged from 59% ± 1.2% to 68% ± 2.2% for all investigated upper airway models (including the Ph. Eur. inlet) and flow rates. These values were far below the labeled dose. A change in flow rate from 30 L/min to 60 or 75 L/min resulted in no further increase of emitted dose in all investigated impactor inlets. 

Regarding the oropharyngeal deposition, a tendency of lower deposition values in the idealized adult model compared to the idealized pediatric upper airway model and the Ph. Eur. inlet could be observed. But due to the partially high standard deviations, no differences in oropharyngeal deposition between the three investigated impactor inlets could be shown. Each deposition determination led to high values for oropharyngeal deposition and comparatively low amount of particles passing the airway model. These results were transferable to data described in the literature investigating a low to medium resistant multidose DPI [27] and they could be explained by inadequate powder deaggregation caused by the low resistance and the insufficient force of the drug delivery device. 

The pulmonary deposition showed no influence induced by the oropharyngeal deposition and the emitted dose. The idealized pediatric upper airway model resulted in a lower pulmonary deposition compared to the Ph. Eur. inlet and the idealized adult model (except Ph. Eur. inlet using 30 L/min). At a steady flow rate of 60 L/min, a higher pulmonary deposition was achieved for the Ph. Eur. inlet (13% ± 0.9%) and the idealized adult upper airway model (15% ± 0.4%) compared to the idealized pediatric model (9% ± 0.4%). By increasing the steady flow rate from 30 to 75 L/min an enhancement of the pulmonary deposition could be obtained. The Ph. Eur. inlet showed the highest increase from 7% ± 0.7% to 14% ± 1.1%. The idealized adult upper airway model exhibited a rise from 11% ± 0.4% to 15% ± 0.5%. The pulmonary deposition using the idealized pediatric model was slightly increased from 9% ± 0.4% to 11% ± 1.1%. This relies on stronger deaggregation processes as consequence of higher energy values caused by higher flow rates. 

### 3.3. Deposition Measurements—Time-Varying Inhalation Profile

The deposition measurements under steady conditions resulted in a low, flow-dependent pulmonary deposition. In the case of the Cyclohaler^®^, this observation was reviewed by a time-varying inhalation profile of a 4 years old boy, shown in Figure 4. Furthermore, the *in vitro* testing was extended by using a realistic pediatric upper airway model.

**Figure 6:**
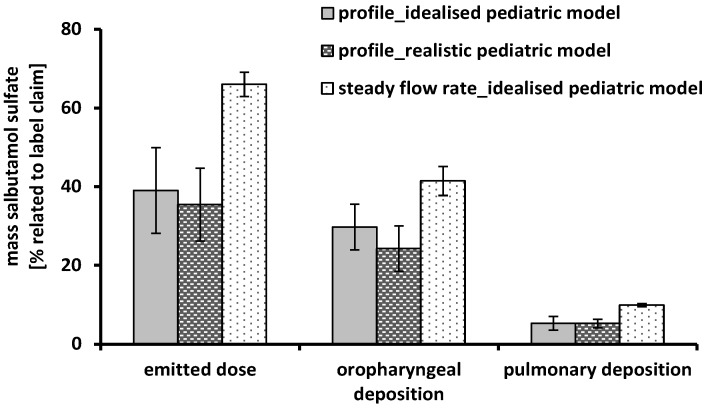
Performance of the Cyclohaler^®^ using the idealized pediatric upper airway model, the realistic pediatric upper airway model, the time-varying inhalation profile (grey and dashed black column) and a steady flow rate of 60 L/min (scored white column); emitted dose, oropharyngeal deposition and pulmonary deposition were related to label claim; mean ± SD, *n* = 3.

Figure 6 shows the performance results of the Cyclohaler^®^ obtained by simulating the inhalation profile in the upper airway model and the inhalation device. No differences between the realistic and the idealized pediatric upper airway model could be detected. Despite of the more detailed structure of the realistic model, the oropharyngeal deposition was comparable. In both cases the pulmonary deposition was about 5% related to label claim. The idealized pediatric upper airway model seemed to be sufficient for *in vitro* DPI testing and led to feasible results.

Another part of the present investigations was the comparison of steady flow rates against the time-varying inhalation profile. Regarding the emitted dose, a high difference between the two inhalation conditions could be observed. The amount of delivered API was 39% ± 10.9% using the inhalation profile and 66% ± 3.1% with the steady flow rate of 60 L/min. These results could possibly be explained by the different flow rates achieved in the DPI and the upper airway models accompanied by an additionally inhomogeneity proceed of the flow rate during the inhalation of the child. The maximum PIF was reached only for a short time of about 0.1 s and led to an insufficient and varying deflation of the capsules incorporated in the device. The oropharyngeal deposition was slightly higher under steady conditions (41% ± 3.7%) compared to the inhalation profile (30% ± 5.8%) and is mainly generated by a flow-dependent high intensity of particle impaction. However, this effect could be counterbalanced by a higher amount of particles smaller than 5 μm (pulmonary deposition) in the case of the steady flow rate. Despite of equal conditions in the NGI (steady flow rate of 60 L/min), there was an increase in pulmonary deposition from 5% ± 1.8% up to 10% ± 0.4% by using a steady flow rate of 60 L/min instead of the inhalation profile (maximum PIF of 30 L/min). The main reasons for this finding has been the enhanced deaggregation of the powder particles primarily occurring in DPI as result of the higher flow rate [34,35] and the much higher emitted dose for steady flow rates.

### 3.4. Transfer to Further DPIs

The results achieved from performance testing of the Cyclohaler^®^ were transferred to two further DPIs, Handihaler^®^ and Spinhaler^®^. The measurements were limited to steady flow rates (30 and 60 L/min) and the idealized adult and pediatric upper airway models.

**Figure 7:**
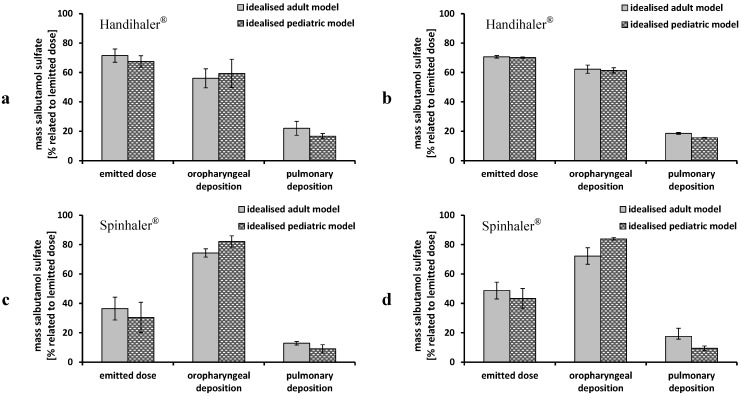
Performance of Handihaler^®^ and Spinhaler^®^ using the idealized adult (grey columns) and pediatric (dashed black columns) upper airway models and steady flow rates; (**a**) Handihaler^®^ 30 L/min; (**b**) Handihaler^®^ 60 L/min; (**c**) Spinhaler^®^ 30 L/min; and (**d**) Spinhaler^®^ 60 L/min; emitted dose, oropharyngeal deposition and pulmonary deposition were related to emitted dose; mean ± SD, *n* = 3.

Figure 7 shows the emitted dose, oropharyngeal and pulmonary deposition obtained by performance testing of the Handihaler^®^ and the Spinhaler^®^. In the case of the Handihaler^®^ no differences between the adult and the pediatric upper airway model could be determined. There was only a slight decrease from 19% ± 0.6% to 16% ± 0.3% in pulmonary deposition using the pediatric model and 60 L/min as steady flow rate. These results were comparable to data obtained by the Cyclohaler^®^. In this context, the device resistance and the pressure drop within the devices (Table 1) has to be taken into account. The Handihaler^®^ is assigned to high resistant DPIs, demonstrated by the fact that a flow rate of only about 40 L/min was needed to cause a pressure drop of 4 kPa within the device [29]. In the case of the Cyclohaler^®^ more than 100 L/min were necessary. This led to various energy inputs, which was closely related to the oropharyngeal and pulmonary deposition. The Handihaler^®^ performance was not affected by flow rate; only the standard deviations were higher using a flow rate of 30 L/min. The Spinhaler^®^ showed low values for the emitted dose. More than half of the powder remained in the capsules and led to high variations of the deposition pattern. This may be due to the exchange of the formulation, which has not been adapted for the device. Compared to the other investigated devices, the Spinhaler^®^ showed the worst performance expressed as low emitted dose, high amount of API deposited in the upper airway models (72%–85% related to emitted dose) and low pulmonary deposition. Differences between the adult and pediatric upper airway model were less distinctive using low flow rates (30 L/min). At a flow rate of 60 L/min the pulmonary deposition in the pediatric model (9% ± 1.5%) was lower than in the adult model (18% ± 1.9%).

Recently reported innovations in the field of aerosol delivery were dealing with changes in the inhaler geometry (mouth piece or capsule chamber) by including a three-dimensional rod array [36,37]. This technology showed an improvement in inhaler performance of several devices, like the here investigated Handihaler^®^ [38].

## 4. Conclusions

The majority of respiratory diseases demand a pulmonary administration of APIs to patients of all age groups. Hence, the therapy should consider age-specific characteristics of the main affecting elements: patient, device and formulation. This research focused on the influencing factors caused by the patient and the drug delivery device. Therefore, a time-varying inhalation profile was recorded during inhalation using the Cyclohaler^®^. It showed a maximum PIF of 30 L/min, which was lower than flow rates described in literature [28,33]. For the Cyclohaler^®^, comparative *in vitro* studies were performed using the pediatric inhalation profile and steady flow rates to investigate the influence of different inhalation conditions. This resulted in a lower pulmonary deposition of 5% ± 1.8% using the inhalation profile compared to 10% ± 0.4% achieved with steady flow rates. Time-varying inhalation profiles are promising tools to obtain information about the inhalation behavior of the patient and could be used for adjusting *in vitro* investigations to special age groups, especially children. However, this study is limited by the fact that only one trained 4 years old child was included. Apart from inhalation parameters influencing the delivery of inhalable particles, the age-specific anatomy of the oropharynx together with the amount of API particles concentrated in this region play an essential role. These particles are not available for lung therapy. In this context, improved upper airway models were included in this study. Using the Cyclohaler^®^, the idealized adult upper airway model led to similar results as the conventional induction port method of the European Pharmacopeia, but represents the upper airway geometry of adults in more detail and is today a tool frequently used for assessing device performance. With respect to children, an idealized and a realistic upper airway model was investigated, and both showed similar pulmonary deposition of about 5% related to label claim. The realistic pediatric upper airway model provided no additional information. There were slight differences manifested between the idealized adult and pediatric model, while the pulmonary depositions achieved using the pediatric model were always lower. The idealized pediatric upper airway model ensured a good reproducibility, confirming the results obtained by Below *et al.* [27]. 

To assess the performance of the Cyclohaler^®^, the flow dependent change in pulmonary deposition has to be taken into account, as this is an important aspect in inhalation therapy. Diversified flow rates, often appearing in children depending on their mental capacity and emotional state, yield an intra-individual heterogeneous therapy including varying amounts of API reaching the peripheral airways. Finally, the Cyclohaler^®^ offered a restricted robustness with regard to varying flow rates. The high resistant device Handihaler^®^ was less affected by flow rate, but led to deposition data comparable to the Cyclohaler^®^. The Spinhaler^®^ showed a comparatively worse performance and limited reproducibility.

With focus on recent newly established requirements of the EMA and FDA regarding pediatric medicines, DPI performance testing in children will gain importance. Therefore, this idealized pediatric upper airway model can be recommended for *in vitro* testing of DPIs with single dosed capsules and is a promising tool in inhaler- and formulation development. 
